# Comprehensive management of ischemic stroke with traditional Chinese medicine: a new exploration

**DOI:** 10.3389/fmed.2026.1765941

**Published:** 2026-03-17

**Authors:** Qianshi Zhang, Xu Dong, Hong Huo, Ying Zhang, Mengyun Zhang, Dongyan Wang

**Affiliations:** 1 Department of Graduate School, Heilongjiang University of Chinese Medicine, Harbin, Heilongjiang, China; 2 Third Ward of Acupuncture and Moxibustion Department, The Second Affiliated Hospital of Heilongjiang University of Chinese Medicine, Harbin, Heilongjiang, China

**Keywords:** diagnosis and treatment, integrative medicine (TCM + modern medicine), ischemic stroke, pathological mechanism, rehabilitation and prevention, TCM

## Abstract

Ischemic stroke exhibits considerable variability across regions, age groups, sexes, and ethnicities, with risk factors encompassing genetic, environmental, and socioeconomic dimensions. Its pathophysiology is multifaceted and involves disturbances in energy metabolism, disruption of the BBB, and inflammatory processes, among other mechanisms. Diagnostic advancements including imaging modalities, biomarker identification, and artificial intelligence applications have significantly enhanced clinical practice. Therapeutic strategies primarily focus on reperfusion and antiplatelet therapies, while traditional Chinese medicine (TCM) demonstrates potential through multi-targeted and multi-modal approaches. Rehabilitation and prevention efforts underscore the importance of multidisciplinary collaboration, personalized treatment plans, and early risk factor interventions. Rooted in the traditional conceptualization of stroke, TCM exerts its effects via anti-inflammatory, antioxidant, and metabolic regulatory pathways, and has shown promising clinical outcomes when integrated with conventional medicine. Nonetheless, challenges remain regarding the robustness of the efficacy evidence, standardization of syndrome differentiation, and safety concerns. Future directions point toward the integration of multi-omics technologies, artificial intelligence, precision medicine, and international collaboration to advance the comprehensive management of ischemic stroke by incorporating TCM.

## Introduction

Ischemic stroke is a cerebrovascular disorder characterized by a high incidence rate, complex risk factors, and diverse pathological mechanisms that pose a significant threat to human health. This study provides a systematic review of the epidemiological features, pathophysiological processes, diagnostic methodologies, and therapeutic approaches associated with ischemic stroke. Particular emphasis is placed on foundational theories, mechanisms of action, and clinical applications of traditional Chinese medicine (TCM) for treating this condition, including its integration with contemporary medical practices. Additionally, this paper critically examines issues related to the efficacy, safety, and standardization of TCM treatments. Finally, it explores future directions emphasizing interdisciplinary collaboration, intelligent technologies, precision medicine, and internationalization, thereby offering a comprehensive theoretical and practical framework for holistic management of ischemic stroke through TCM.

## Methods of literature search

We conducted a narrative literature search. This article is a narrative review in PubMed, Web of Science, Embase, and CNKI from January 2010 to December 2025. Search terms included combinations of “ischemic stroke,” “traditional Chinese medicine,” “integrative medicine,” “Chinese herbal formula,” “TCM injection,” “acupuncture,” “rehabilitation,” “ferroptosis,” “exosome/extracellular vesicles,” “gut–brain axis,” and “network pharmacology.” We preferentially included English-language peer-reviewed articles, multicenter randomized controlled trials, and meta-analyses/systematic reviews, as well as mechanistic studies with experimental validation. Lower-level evidence was used only to illustrate emerging hypotheses.

### Epidemiology

The epidemiological profile of ischemic stroke shows substantial geographic, sex-, and age-related disparities. Recent Global Burden of Disease (GBD) 2021 analyses indicate that ischemic stroke constitutes the largest proportion of incident strokes worldwide and that absolute incident cases have continued to rise, with several regions showing a stagnation in the decline of age-standardized rates since around 2015 (1–3).

Risk factors associated with ischemic stroke involve a complex interplay of genetic, environmental, and socioeconomic determinants. Recent large-scale integrative genome-wide association analyses have expanded the catalog of ischemic stroke–associated loci and implicated pathways related to cerebrovascular function, inflammation, metabolism, and iron handling (4). Among environmental contributors, the interaction between tobacco smoking and the prothrombin G20210A polymorphism was found to substantially increase the risk of cryptogenic stroke (5). Socioeconomic factors also play a critical role. A population-based study in South London demonstrated that higher levels of socioeconomic deprivation were significantly associated with poorer functional recovery following stroke, particularly among elderly women and ischemic stroke patients without prior comorbid conditions (6). Additionally, a reduced estimated glomerular filtration rate (eGFR < 30 mL/min/1.73 m^2^) was identified as an independent predictor of unfavorable functional outcomes 3 months after post-ischemic stroke (7).

### Pathophysiological mechanisms of ischemic stroke

The pathophysiology of ischemic stroke encompasses intricate cellular and molecular processes. Following cerebral ischemia, disruptions in energy metabolism result in the failure of ion pump function, subsequently initiating excitotoxicity mediated by amino acids, oxidative stress, and inflammatory responses. A metabolomic analysis of young ischemic stroke patients revealed significant modifications in serum metabolic profiles, particularly involving arginine biosynthesis, glycerophospholipid metabolism, and the taurine and hypotaurine metabolic pathways (8). Mitochondrial dysfunction is a critical contributor to ischemic brain injury. Research indicates that the transport of p53 between the mitochondria and the nucleus modulates neuronal susceptibility to cerebral ischemia, with mitochondrial localization of p53 being closely associated with apoptotic processes (9). Furthermore, the interplay between endoplasmic reticulum (ER) stress and mitochondrial dysfunction, notably via mitochondria-ER contact sites, exacerbates ischemic brain damage (10). A recent comprehensive review further summarized experimental evidence that multiple TCM modalities (herbal prescriptions, extracts, and acupuncture) may mitigate ischemic brain injury by preserving mitochondrial structure/function and attenuating mitochondrial-driven oxidative stress and apoptosis (11).

Disruption of the blood-brain barrier (BBB) constitutes a pivotal event in the pathophysiological cascade of ischemic stroke. Hyperglycemia exacerbates hemorrhagic transformation after ischemic stroke, a process mediated by oxidative stress, inflammatory responses, and activation of matrix metalloproteinases (MMPs) (12). The Hsp90 inhibitor 17-DMAG has demonstrated efficacy in mitigating hyperglycemia-induced hemorrhagic transformation by suppressing inflammation and MMP activity (12). Additionally, dysfunction of the gut barrier contributes to ischemic stroke pathophysiology through the gut-brain axis, which is characterized by alterations in intestinal microbiota, increased intestinal permeability, and bacterial translocation (13). Notably, the levels of C-terminal proendothelin-1 (CT-pro-ET-1) peak 1 day following ischemic stroke and independently predict 90-day mortality, exhibiting a prognostic value superior to that of traditional risk factors (14).

Recent advances have significantly enhanced our understanding of the molecular regulatory networks underlying the pathology of ischemic stroke. MicroRNAs (miRNAs) are key post-transcriptional regulators that play essential roles in the development of stroke. For instance, miR-126, miR-17-92 cluster, and miR-155 are implicated in atherogenesis, whereas miR-145, miR-146, and miR-217 influence carotid plaque progression and stability (15). During acute stroke, dynamic expression patterns of miRNAs such as let-7, miR-21, miR-29, miR-124, miR-145, miR-181, miR-210, and miR-223 are closely associated with cerebral injury and repair mechanisms (15). Moreover, epigenetic modifications, including DNA methylation, histone modifications, and regulation by non-coding RNAs, play significant roles in ischemic stroke pathogenesis (16).

Inflammation exerts a dual influence on the pathophysiology of ischemic stroke. It aggravates cerebral injury, which is characterized by microglial activation, release of proinflammatory cytokines, and neutrophil infiltration. Inflammatory processes also contribute to tissue repair and regeneration. Studies have identified the cGAS-STING signaling pathway as a mediator of inflammatory responses in ischemic stroke through regulation of microglial M1/M2 polarization, and inhibition of this pathway has been shown to reduce cerebral infarct volume and improve neurological outcomes (17). Additionally, ischemia-reperfusion injury remains a significant therapeutic challenge, with the underlying mechanisms involving oxidative stress, calcium overload, mitochondrial dysfunction, and inflammation (18). Recent therapeutic developments, such as hypoxia-reperfusion strategies, have demonstrated the potential to attenuate oxidative stress-induced injury (19).

### Diagnosis

Recent advancements in diagnostic methodologies for ischemic stroke are particularly notable in the domains of imaging and biomarker identification. Techniques such as Computed Tomography Perfusion Imaging (CTP) and Magnetic Resonance Perfusion Imaging (MRP) enable early detection of perfusion abnormalities within ischemic cerebral tissue, thereby providing critical information to guide clinical decision-making processes (20). The integration of artificial intelligence (AI) in ischemic stroke diagnosis is progressively expanding; for instance, the Shukun AI software demonstrates an accuracy rate of 86.4% in evaluating ischemic penumbra and core infarction, although a margin of 13.6% inaccuracy persists (21). Furthermore, high-resolution vascular wall imaging (HR-VWI) offers precise visualization of the location and characteristics of the vascular stenosis. When combined with apolipoprotein E (APOE) genotyping, HR-VWI enhances both diagnostic precision and prognostic evaluation in cases of acute ischemic stroke (22).

Significant progress has also been made in the utilization of biomarkers for the diagnosis of ischemic stroke. Elevated activity of lipoprotein-associated phospholipase A2 (Lp-PLA2-A) has been strongly linked to the pathophysiology of artery-to-artery embolization in symptomatic intracranial atherosclerotic disease (sICAD), with higher levels correlating positively with an increased incidence of cortical infarcts (23). Additionally, biomarkers such as high-sensitivity C-reactive protein (hs-CRP), serum ferritin, and high-density lipoprotein (HDL) have demonstrated utility in differentiating between ischemic and hemorrhagic stroke (24). Investigations into the microbiome have revealed heterogeneity in the microbial communities associated with cerebral thrombosis of varying etiologies, offering novel perspectives for the etiological diagnosis of ischemic stroke (25). Nonetheless, current diagnostic approaches face challenges, including difficulties in distinguishing internal carotid artery (ICA) dissection from carotid webs via ultrasound evaluation (26) and complications arising from contrast media extravasation (27).

Emerging technological trends in ischemic stroke diagnosis are anticipated to center on innovations in imaging modalities, biomarker development, and the expanded application of artificial intelligence. Advances in imaging, particularly HR-VWI and perfusion imaging, are expected to yield more precise data for the early diagnosis and etiological differentiation of ischemic stroke (28). For example, HR-VWI can delineate detailed vessel wall features, facilitating the identification of atherosclerotic plaque characteristics (28). In the realm of biomarkers, the deployment of multi-omics technologies promises to uncover additional biomarkers pertinent to ischemic stroke (29), with gut microbiome analyses identifying several microbial markers linked to the condition (30).

Artificial intelligence is projected to play an increasingly prominent role in the diagnosis of ischemic stroke (31). Machine-learning-based diagnostic models capable of integrating multimodal data have been poised to enhance diagnostic accuracy and prognostic evaluations (31). Moreover, the advancement of mobile health technologies and remote diagnostic tools is expected to provide more accessible means for early detection of ischemic stroke (32). Realization of these future technological developments will necessitate multidisciplinary collaboration and sustained innovation (29).

## Treatment strategy

### Acute-phase management in modern medicine

Reperfusion therapy has advanced substantially, and mechanical thrombectomy is a standard of care for ischemic stroke with large-vessel occlusion. The ESCAPE trial reported a reduced median infarct volume with thrombectomy compared with control treatment (33), and RevASCAT showed improved 90-day functional outcomes with reduced early neurological deficits assessed by the National Institutes of Health Stroke Scale (NIHSS) (34). For malignant middle cerebral artery infarction, partial stroke resection combined with cisternal cerebrospinal fluid drainage has been reported as a safe and effective intervention with lower complication rates (35).

### Secondary prevention and medical management in modern medicine

Antiplatelet therapy remains pivotal for many patients. The ARAMIS trial reported non-inferiority of dual antiplatelet therapy (DAPT) versus intravenous thrombolysis in mild, non-disabling acute ischemic stroke, with favorable 90-day functional outcomes (modified Rankin Scale 0–1) (36). The ATAMIS trial further showed that clopidogrel plus aspirin was superior to aspirin alone in reducing early neurological deterioration within 7 days in mild-to-moderate acute ischemic stroke (37).

### TCM therapies and evidence landscape

Traditional Chinese medicine has been used as a multi-modal intervention across acute to recovery stages. For example, Buyang Huanwu Decoction administered alongside conventional treatment has been reported to improve neurological function and activities of daily living in patients with ischemic stroke (38). However, the evidence base is heterogeneous, and many studies remain small or single-center; therefore, benefits should be interpreted in the context of trial quality, standardization (including syndrome differentiation), and safety reporting. Syndrome differentiation (pattern-based stratification) does not represent a one-to-one correspondence with biomedical categories. For international trial design, pattern classification should be operationalized using predefined diagnostic criteria combined with standardized neurological scales and selected laboratory or imaging markers where appropriate. Common patterns and pragmatic anchors for standardization are summarized in Table 1.

**TABLE 1:** Common traditional Chinese medicine (TCM) patterns in ischemic stroke and pragmatic anchors for trial operationalization.

TCM pattern (English)	Typical features (very brief)	Pragmatic biomedical anchors (examples)	Trial operationalization (examples)
Qi deficiency + blood stasis	Fatigue; chronic deficits	Poor perfusion/endothelial dysfunction	Pattern checklist + NIHSS/mRS; document perfusion imaging if available
Phlegm-heat	Heaviness; constipation; irritability	Inflammation/metabolic burden	CRP (if available) + glucose/lipids; predefined symptom checklist
Wind-phlegm/channel obstruction	Prominent motor/speech deficits	Edema / neuroinflammation (contextual)	NIHSS domains + imaging edema markers (when available)
Blood stasis predominance	Fixed symptoms; dark/purple tongue (TCM)	Platelet activation/microcirculation issues	Concomitant antithrombotics recorded; bleeding monitoring plan
Yin deficiency–yang hyperactivity	Dizziness; insomnia	Autonomic dysregulation/stress response	Sleep scale + BP variability (if collected); fixed assessment timepoint
Heat-toxin/excess heat (optional)	Restlessness; “heat” signs	Acute inflammatory surge/infection confounding	Infection screen; avoid conflating stroke complications with pattern

These anchors are not one-to-one equivalence with biomedical diagnoses; they are pragmatic elements to improve reporting and standardization in integrative trials.

### Combination therapy (TCM + modern medicine): scenarios, synergy, and safety

Beyond parallel use, combination therapy in ischemic stroke aims to integrate guideline-based reperfusion and antithrombotic management with adjunct TCM interventions across the acute-to-recovery continuum, with the goals of mitigating ischemia–reperfusion (I/R) injury and supporting secondary prevention and rehabilitation. It should be noted that some neuroprotective combinations (e.g., mechanical thrombectomy plus selective intra-arterial hypothermia) have also been explored within modern medicine to attenuate reperfusion-related injury (39).

**FIGURE 1:**
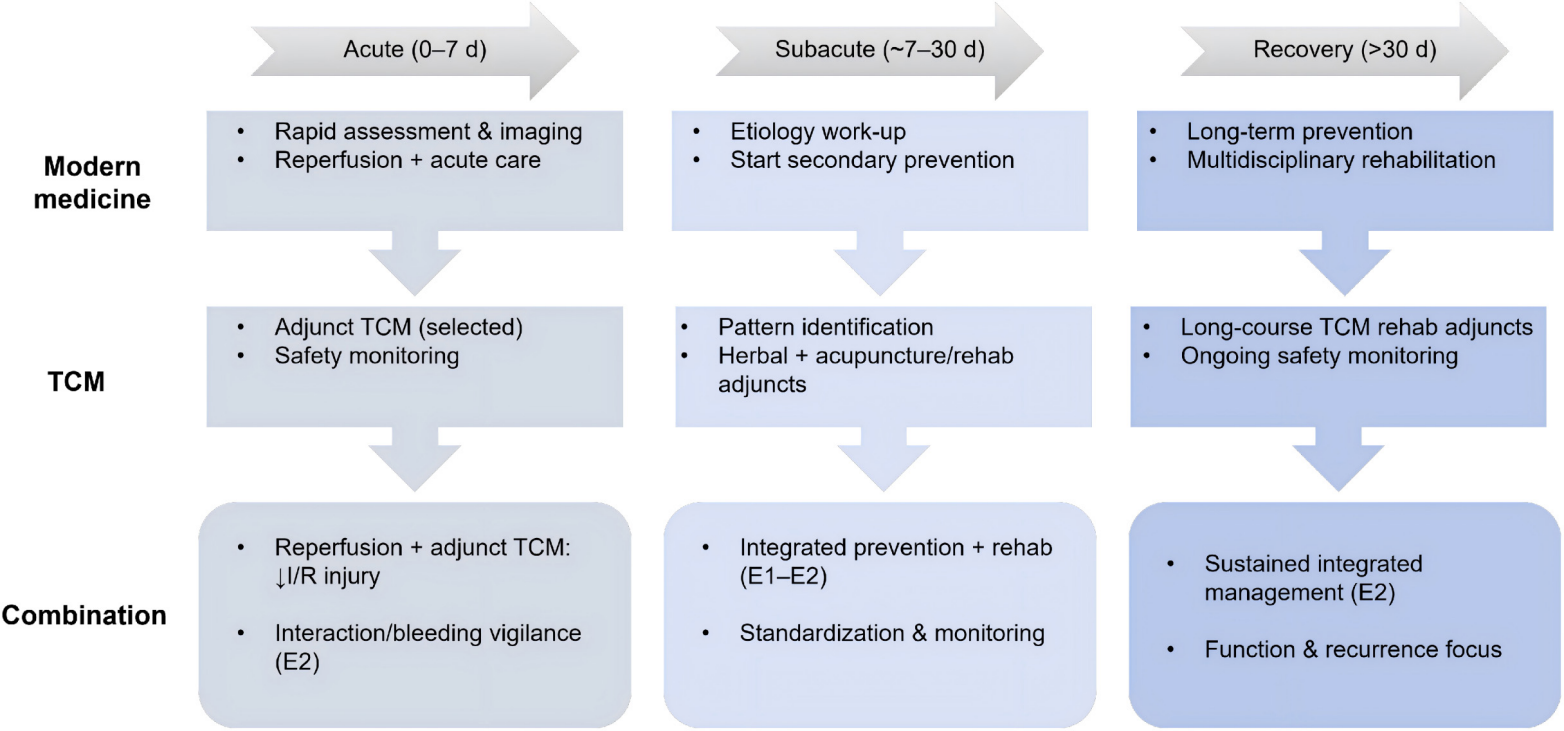
Integrated TCM–modern medicine pathway for comprehensive management of ischemic stroke. The framework summarizes phase-specific management across the acute (0–7 days), subacute (∼7–30 days), and recovery/rehabilitation (>30 days) stages, distinguishing modern medicine, TCM, and their combination. Combination therapy highlights potential synergy of reperfusion with adjunct TCM to mitigate ischemia–reperfusion (I/R) injury and to support integrated secondary prevention and rehabilitation, with emphasis on safety monitoring and standardization. Evidence level is denoted as E1 (multicenter RCT/meta-analysis) and E2 (small or single-center clinical studies). TCM, traditional Chinese medicine; I/R, ischemia–reperfusion; RCT, randomized controlled trial; Symbol/arrow meaning: “↓” indicates reduction/attenuation (e.g., ↓ I/R injury).

When TCM is used as an adjunct to modern medicine, the key practical considerations include (i) clear clinical scenarios and timing (acute/subacute/rehabilitation), (ii) evidence level (E1–E2) and risk of bias, and (iii) safety monitoring, particularly potential herb–drug interactions, bleeding risk under concomitant antiplatelet/anticoagulant therapy, and product quality control for herbal products/injections. Representative adjunct TCM options, proposed mechanisms, evidence levels, and major limitations/safety notes are summarized in Table 2. The overall phase-specific integrated pathway for TCM–modern medicine management is summarized in Figure 1. Recent integrative syntheses also discuss potential synergies when acupuncture and TCM herbal medicine are combined with conventional approaches in stroke management (40).

**TABLE 2:** Representative traditional Chinese medicine (TCM) interventions in ischemic stroke: mechanisms and evidence level.

Intervention	Clinical scenario (add-on)	Key mechanism (examples)	Evidence level*	Key limitations
Buyang Huanwu decoction	Subacute–recovery	Anti-inflammatory; oxidative stress modulation; possible anti-ferroptosis axis	E2 (±P)	Heterogeneous protocols; need multicenter RCTs
Danhong injection	Acute–subacute	Endothelial protection; microcirculation improvement	E2	Injection safety; bleeding risk monitoring
Xuesaitong (notoginseng saponins)	Acute–subacute	Anti-platelet-related signaling; BBB protection	E2	Interaction with antithrombotics
Acupuncture	Subacute–rehabilitation	Neuroplasticity; perfusion modulation	E1/E2 (context-dependent)	Protocol variability; blinding difficulty
Ferroptosis-related TCM mechanisms	Mechanistic rationale	Nrf2–GPX4 axis; lipid peroxidation suppression	P	Mostly preclinical; translational gap

*E1, multicenter RCT/meta-analysis; E2, small/single-center clinical study; P, preclinical only.

## Rehabilitation and preventive measure of ischemic stroke

In recent years, rehabilitation strategies for ischemic stroke have increasingly prioritized multidisciplinary collaboration and personalized treatment plans. Nutritional status plays a crucial role in determining the prognosis of stroke patients. The Prognostic Nutrition Index (PNI) has been found to be significantly correlated with in-hospital mortality, with patients exhibiting a PNI greater than 37.45 demonstrating higher survival rates (41). Post-stroke dysphagia is common and is a major contributor to aspiration pneumonia, malnutrition, and adverse functional outcomes; therefore, early screening and targeted interventions are essential, especially in older adults (42). Virtual reality (VR)–based interventions have been reported to improve selected post-stroke affective or cognitive–emotional outcomes (including alexithymia), although the evidence remains preliminary and heterogeneous (43). Additionally, advancements have been made in the application of TCM within rehabilitation contexts; for instance, Buyang Huanwu Decoction has been reported in preclinical models to mitigate ischemic brain injury by inhibiting ferroptosis, a form of iron-dependent cell death (44).

Preventive measures for ischemic stroke primarily focus on management of risk factors and antithrombotic therapy. Direct oral anticoagulants (DOACs) have demonstrated superiority in stroke prevention among patients with atrial fibrillation, offering a reduced risk of ischemic stroke and fewer bleeding complications than warfarin (45). In patients with cryptogenic stroke, DOACs did not show a significant advantage over aspirin in preventing recurrence; however, they provided some protective benefits in individuals aged ≥ 75 years (45). Moreover, lifestyle modifications, including smoking cessation, weight management, and glycemic control, are essential for ischemic stroke prevention (46). Notably, individuals with type 2 diabetes exhibit an elevated risk of cardiovascular events up to 30 years prior to diagnosis, underscoring the importance of implementing preventive strategies at an earlier stage (47).

## Basic theory of comprehensive treatment of ischemic stroke with TCM

Ischemic stroke is classified under the category of “apoplexy” within the TCM framework. Its etiology and pathogenesis are multifaceted and primarily involve factors such as wind, fire, phlegm, blood stasis, and deficiency. According to the TCM theory, the onset of apoplexy is predominantly attributed to a deficiency of vital qi, dysfunction of the internal organs, emotional disturbances, improper diet, and irregular work-rest patterns. These factors collectively result in the disruption of qi and blood flow, leading to cerebral vascular obstruction or extravasation of blood, thereby causing the disease.

In recent years, the integration of modern research methodologies has facilitated significant advancements in the understanding of the etiology and pathogenesis of ischemic stroke from a TCM perspective. For instance, puerarin, a bioactive compound derived from TCM, has been shown to protect brain tissue against ischemia–reperfusion injury by inhibiting inflammatory responses, potentially mediated by downregulation of Toll-like receptor 4, myeloid differentiation factor 88, nuclear factor-κB, and tumor necrosis factor-α (48). In parallel, ferroptosis and iron homeostasis have been increasingly recognized as actionable injury nodes in ischemic stroke, and recent data-mining/network analyses highlight ferroptosis-regulatory targets as convergent mechanisms across commonly used TCM interventions (49). Furthermore, the combined administration of Astragalus membranaceus extract and Panax notoginseng saponins enhances protection against cerebral ischemia–reperfusion injury by improving energy metabolism and inhibiting apoptosis, with regulation of adenosine triphosphate levels, Na^+^-K^+^ ATPase activity, and mitochondrial apoptotic pathways (50). Collectively, these findings suggest that TCM concepts such as “Qi and blood stasis” and “phlegm retention” can map onto contemporary biomedical mechanisms including inflammatory signaling, energy metabolism disturbance, and iron-dependent lipid peroxidation.

Further investigations have revealed that Huanglian Jiedu Decoction exerts therapeutic effects on ischemic stroke through multi-component and multi-target actions, with recent multi-omics evidence supporting modulation of neuroinflammation and programmed cell death pathways such as pyroptosis (51). Notably, exosome/extracellular vesicle–related advances have emerged as a new frontier for TCM research: Panax notoginseng–derived exosome-like nanoparticles have been reported in experimental models to attenuate cerebral ischemia–reperfusion injury by altering microglial polarization and improving blood–brain barrier integrity (52), and Houttuynia cordata–derived extracellular vesicle-like particles were shown in experimental models to alleviate ischemic brain injury via miRNA-mediated suppression of ferroptosis-related targets (53). These studies illustrate how multi-component TCM interventions and TCM-derived vesicular carriers may converge on key injury programs in ischemic stroke.

**FIGURE 2:**
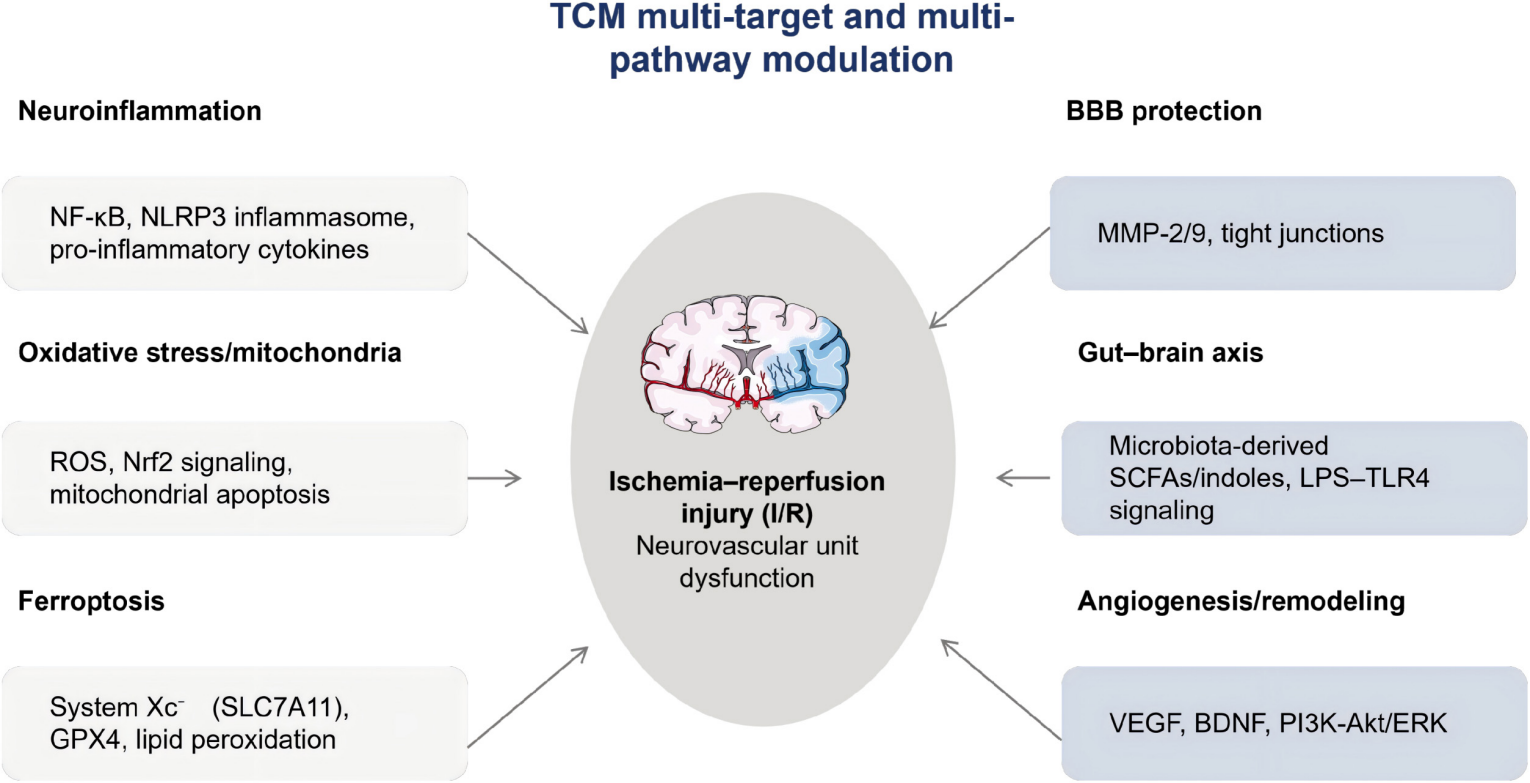
Multi-target mechanisms by which traditional Chinese medicine (TCM) modulates ischemic stroke pathology. This schematic illustrates key molecular and cellular pathways involved in ischemic stroke, converging on ischemia–reperfusion (I/R) injury and neurovascular unit dysfunction. TCM is depicted as a multi-target, multi-pathway modulator acting on neuroinflammation, oxidative stress and mitochondrial dysfunction, ferroptosis, blood–brain barrier (BBB) integrity, gut–brain axis signaling, and angiogenesis/remodeling. Rather than indicating single-target effects, the figure emphasizes the network-based regulatory characteristics of TCM and its potential synergy with modern medicine in mitigating I/R injury and promoting functional recovery.

However, such pathway annotations (e.g., “GPCR signaling”) are often derived from network-based enrichment analyses rather than receptor-level validation; therefore, they should be interpreted as hypothesis-generating links to downstream inflammatory/oxidative axes (e.g., PI3K-Akt/MAPK-NF-κB) that require targeted experimental confirmation (Figure 2) (54, 55).

The theoretical framework guiding TCM interventions for ischemic stroke is rooted in the holistic philosophy of TCM and the principle of syndrome differentiation and treatment, emphasizing the dual objectives of “strengthening body resistance and eliminating pathogens” as well as addressing both symptomatic manifestations and underlying causes. Recent advances in network pharmacology, molecular biology, and related technologies have expanded this theoretical basis. For example, Buyang Huanwu Decoction, a classical TCM formulation, mediates its therapeutic effects on ischemic stroke through multi-component and multi-target actions, regulating pathways such as calcium signaling, vascular smooth muscle contraction, and nucleotide-binding oligomerization domain-like receptor signaling (56). Additionally, the Wendan Decoction has demonstrated efficacy in treating both ischemic and hemorrhagic stroke, improving neurological deficit scores and overall treatment outcomes (57).

Contemporary research has substantiated that the theoretical basis of TCM treatment for ischemic stroke extends beyond the traditional concepts of “Qi, blood, and body fluid,” encompassing mechanisms recognized in modern medicine, including neuroprotection, anti-inflammatory effects, and antioxidation. For instance, Naoxintong capsule confers neuroprotection against cerebral ischemia-reperfusion injury by downregulating the expression of lectin-like oxidized low-density lipoprotein receptor 1, phosphorylated ERK1/2, and nuclear factor κB (58). Furthermore, electroacupuncture (EA) pretreatment mitigates cerebral ischemia-reperfusion injury by activating the nuclear factor E2-related factor 2 pathway and suppressing oxidative stress and inflammatory responses (59). These findings validate the molecular basis of TCM therapeutic principles for ischemic stroke and provide a robust scientific rationale for their clinical application. Key multi-target mechanisms discussed in this section are summarized in Figure 2.

## Effect of TCM on pathological mechanism of ischemic stroke

The therapeutic mechanisms of TCM in ischemic stroke encompass multiple facets, including anti-inflammatory, antioxidative, and anti-apoptotic effects and the promotion of angiogenesis. For instance, puerarin confers neuroprotection against ischemia-reperfusion injury by attenuating inflammatory responses, potentially through the downregulation of Toll-like receptor 4, myeloid differentiation factor 88, nuclear factor-κB, and tumor necrosis factor-α (48). Moreover, the combined administration of total astragalus extract and total saponins from Panax notoginseng has been shown to augment neuroprotection in cerebral ischemia-reperfusion injury by enhancing energy metabolism and inhibiting apoptotic pathways (50). Recent advancements facilitated by network pharmacology and molecular biology techniques have significantly deepened our understanding of the mechanisms of TCM in ischemic stroke treatment. For example, the Huanglian Jiedu Decoction exerts therapeutic effects via a multi-component, multi-target approach, with its active constituents modulating pathways, such as G protein-coupled receptor signaling, neural ligand-receptor interactions, and gap junction communication (51).

Further investigations have revealed that TCM’s efficacy of TCM in ischemic stroke is intricately linked to the regulation of iron metabolism. Specifically, Naotaifang extract has been demonstrated to restore iron homeostasis in the hippocampal CA2 region following cerebral ischemia by modulating the expression of iron transporters, thereby affording neuronal protection (49). Additionally, Danhong injection facilitates angiogenesis and neural repair through upregulation of vascular endothelial growth factor and hypoxia-inducible factor 1α (60). Collectively, these findings underscore the multicomponent and multitarget characteristics of TCM mechanisms in ischemic stroke, providing a robust scientific foundation for its clinical application.

## Clinical practice of treating ischemic stroke with combination of TCM and modern medicine

The clinical application of TCM in the management of ischemic stroke prioritizes syndrome differentiation and individualized therapeutic approaches. Commonly used modalities include compound herbal formulations, acupuncture, and massage therapy. For instance, Buyang Huanwu Decoction has been reported to improve neurological deficits and activities of daily living in ischemic stroke patients (see also section “Basic Theory of comprehensive treatment of ischemic stroke with TCM”) (56). Similarly, Wendan Decoction has exhibited favorable outcomes in treating both ischemic and hemorrhagic stroke (57). Notably, higher-level clinical evidence has also emerged for standardized multi-herb formulations: a large multicenter, placebo-controlled randomized clinical trial reported that Tongxinluo administered within 72 h of symptom onset improved 90-day functional outcomes in patients with acute ischemic stroke (61).

Further investigations have shown that the clinical efficacy of TCM in ischemic stroke is closely associated with disease severity. Qi deficiency and blood stasis syndrome, a prevalent TCM syndrome in ischemic stroke, responds notably to treatment with the Qilong capsule (62). Additionally, the safety and therapeutic benefits of ginkgolide injection in patients with cerebral infarction have been substantiated, with a low incidence of adverse effects (63). These findings underscore the distinctive characteristics of syndrome differentiation in TCM practice and highlight the variability in patient responses contingent on syndrome classification and disease severity.

Individualized comprehensive treatment regimens in TCM emphasize precise syndrome differentiation to tailor therapeutic strategies accordingly. For example, patients diagnosed with qi deficiency and blood stasis syndrome are frequently treated with modified Buyang Huanwu Decoction, whereas those presenting with phlegm-heat fu-organ excess syndrome are treated with modified Xinglou Chengqi Decoction (64). Acupuncture protocols are similarly customized, selecting acupoints such as Baihui, Shuigou, and Neiguan based on the patient’s specific condition and syndrome type (65). Recent advances in network pharmacology and molecular biology have facilitated significant progress in the elucidation of individualized treatment schemes. Network pharmacology studies have identified that active constituents within the Astragalus-Salvia miltiorrhiza drug pair exert therapeutic effects by modulating targets including ADORA2A and ADORA1, thereby providing a mechanistic foundation for individualized treatment planning (66).

Moreover, individualized TCM treatment approaches for ischemic stroke are increasingly being recognized to be influenced by patient-specific genetic polymorphisms. For instance, apolipoprotein E gene polymorphism has been shown to affect patient responsiveness to TCM interventions (67). Metabolomics-based research further indicates that patients with ischemic stroke with distinct syndromes exhibit differential metabolic profiles, offering additional insights for personalized therapeutic design (68). Collectively, these studies suggest that individualized comprehensive TCM treatment for ischemic stroke is progressively advancing toward precision medicine.

The integrative strategy combining TCM and modern medicine in ischemic stroke treatment leverages complementary advantages, such as the concurrent use of adjunct TCM with reperfusion/antithrombotic therapy, as well as the integration of acupuncture with rehabilitation training. A national cross-sectional study reported that approximately 92.6% of hospitalized ischemic stroke patients received TCM interventions, predominantly in the form of TCM injections and patent Chinese medicines (69). Acupuncture, a pivotal component of TCM, has been reported to support functional recovery by modulating cerebral blood flow and neurological function (70). Evidence-based standardization efforts for integrative protocols have been increasingly emphasized (see also section “Standardization”).

Further research indicates that the efficacy of combined TCM and modern medical treatment strategies is also contingent on disease severity. Patients exhibiting qi deficiency and blood stasis syndrome benefit significantly from the addition of the Qilong capsule to conventional therapy (62). Moreover, the combined use of ginkgolide injection with standard treatment in cerebral infarction patients has been validated for both efficacy and safety, with minimal adverse events reported (63). These findings affirm that the integrative therapeutic approach is characterized by syndrome differentiation and tailored treatment, with patient responses varying according to syndrome type and disease severity.

## Controversies on the comprehensive treatment of ischemic stroke with TCM

The debate surrounding the effectiveness of TCM in treating ischemic stroke primarily centers on the quality of clinical evidence and criteria used to assess therapeutic outcomes. For example, a recent systematic review and meta-analysis of randomized controlled trials suggested that acupuncture as an adjunct to conventional care may improve short-term neurological scores and functional measures in acute ischemic stroke, but the certainty of evidence remains low due to heterogeneity and risk of bias (71). Meanwhile, an increasing number of sham-controlled and multicenter randomized studies are being conducted to strengthen the evidence base; for example, a multicenter, sham-controlled randomized clinical trial reported that manual acupuncture improved neurological function in acute cerebral infarction without increasing safety events (72). For multi-component herbal medicines, the field is also moving toward more rigorous trial designs. A large multicenter randomized clinical trial indicated that Tongxinluo could improve 90-day favorable functional outcomes when added to standard care in acute ischemic stroke (61); nevertheless, generalizability beyond specific formulations and settings remains to be established.

Further investigations have revealed that the controversy over TCM’s efficacy of TCM in ischemic stroke treatment is also linked to the lack of standardization in syndrome differentiation and therapeutic approaches. For example, Qi deficiency and blood stasis syndrome are common diagnostic categories in ischemic stroke, yet the diagnostic criteria for this syndrome vary across studies (62). Moreover, the criteria for evaluating efficacy in TCM often differ from those employed in modern medicine; for instance, improvements in TCM syndrome scores may not correspond directly to changes in neurological deficit scores (73). These findings suggest that resolving the debate over TCM’s efficacy of TCM in ischemic stroke requires the establishment of standardized syndrome differentiation, treatment protocols, and efficacy evaluation systems.

Regarding safety, concerns regarding TCM in ischemic stroke treatment primarily involve adverse reactions and potential drug interactions. For example, Angong Niuhuang Wan contains cinnabar and realgar, which may lead to heavy metal accumulation with prolonged use (74). Additionally, certain TCM injections have been associated with allergic reactions and infusion reactions (75). Recently, improvements in pharmacovigilance systems have heightened the attention to the safety profile of TCM in this context. For instance, a systematic review found a low incidence of adverse events associated with Danshen injection in patients with acute ischemic stroke (72), and another review reported similarly low adverse event rates for ginkgolide injection in cerebral infarction patients (63). These studies underscore the necessity for stringent quality control and vigilant monitoring of adverse reactions to ensure the safety of TCM in ischemic stroke treatment.

From a methodological perspective, common limitations across the available clinical literature include small sample sizes, single-center designs, unclear randomization/blinding procedures, heterogeneous background therapies, and inconsistent endpoint selection. In particular, improvements in TCM syndrome scores or short-term neurological scales may not consistently translate into hard outcomes such as long-term functional independence (mRS), mortality, or hemorrhagic transformation rates. Moreover, variability in syndrome differentiation criteria, formula composition/dose, and product quality control can amplify between-study heterogeneity and reduce reproducibility. Therefore, future trials should prioritize protocolized pattern operationalization, harmonized core outcomes (e.g., NIHSS/mRS/ADL with prespecified follow-up), transparent reporting of concomitant antithrombotics, and rigorous safety monitoring.

Further research has highlighted that the safety of TCM is intricately related to drug interactions. For example, concurrent use of TCM with anticoagulants may elevate bleeding risk (76), whereas combining TCM with antiplatelet agents may increase gastrointestinal adverse effects (69). These findings indicate that ensuring the safety of TCM in ischemic stroke requires rational drug compatibility and individualized treatment regimens.

The standardization of TCM in ischemic stroke treatment encompasses the harmonization of syndrome differentiation and therapeutic strategies, development of efficacy evaluation systems, and implementation of quality control measures. For example, variability in the diagnostic criteria for TCM syndrome types in ischemic stroke complicates the comparison of clinical study outcomes (62). Additionally, efficacy evaluation systems in TCM often diverge from those used in conventional medicine, as improvements in TCM syndrome scores may not align with changes in neurological deficit scores (73). With the progression of evidence-based medicine, the challenges of standardizing TCM for ischemic stroke have received increasing attention. For instance, an expert consensus has provided recommendations regarding the clinical use of Qilong capsules for ischemic stroke, including indications, dosage, and precautions (77).

Further studies have identified quality control as a critical component of standardization challenges. For example, quality control standards for TCM injections require enhancements to ensure both safety and efficacy (75). Moreover, given the complex chemical composition of TCM compounds, quality control necessitates the adoption of multi-index detection methods (77). Collectively, these findings indicate that addressing the standardization challenges of TCM in ischemic stroke treatment requires the establishment of standardized syndrome differentiation and treatment protocols, robust efficacy evaluation systems, and rigorous quality control procedures.

## Discussion

The application of TCM in the treatment of ischemic stroke presents extensive research potential, encompassing fields such as multiomics technologies, artificial intelligence, and precision medicine. For instance, investigations utilizing network pharmacology have identified that active compounds within the Astragalus-Salvia miltiorrhiza drug pair exert therapeutic effects by modulating targets, including ADORA2A and ADORA1, thereby providing a foundation for the advancement of precision medicine (66). Moreover, metabolomics-based studies have demonstrated that the bioactive extract of Naodesheng influences pathways associated with energy metabolism, amino acid metabolism, and oxidative stress in rodent models of ischemic stroke, offering novel insights into the mechanistic underpinnings of TCM (68). Recently, the integration of artificial intelligence has propelled TCM research towards intelligent methodologies; for example, deep learning-based tongue diagnosis systems have achieved high accuracy in automatically identifying tongue features characteristic of ischemic stroke patients (78).

Further research indicates that the prospects of TCM for ischemic stroke treatment are intricately linked to stem cell therapy. Specifically, TCM has been shown to facilitate neural repair after cerebral ischemia by regulating stem cell proliferation and differentiation (79). Additionally, nanotechnology-driven studies have revealed that nanoformulations of TCM active ingredients enhance bioavailability and targeting efficiency (80). Collectively, these findings suggest that TCM research on ischemic stroke is progressively evolving into an interdisciplinary domain. Technological innovations in the comprehensive management of ischemic stroke using TCM include the modernization of herbal preparations, advancements in acupuncture techniques, and the development of rehabilitation devices. For example, nanoformulated Naotaifang extract exhibits improved bioavailability and brain-targeting capabilities (49), whereas electroacupuncture has been reported to enhance patient prognosis by modulating cerebral blood flow and neurological function (65). The advent of three-dimensional (3D) printing technology has further facilitated significant progress in TCM-based comprehensive treatment, exemplified by 3D-printed TCM patches that enable precise drug delivery (81).

Technological innovations in TCM for ischemic stroke are increasingly intertwined with artificial intelligence. Machine learning approaches have elucidated the correlations between TCM syndromes and laboratory biomarkers, thereby underpinning the development of individualized therapeutic regimens (73). Furthermore, virtual reality-based rehabilitation equipment, when combined with acupuncture, has demonstrated efficacy in augmenting recovery outcomes in patients with ischemic stroke (82). Collectively, these advancements indicate a trend towards the intellectualization and precision of TCM technologies in comprehensive ischemic stroke treatment.

The internationalization of TCM in ischemic stroke management primarily encompasses collaborative research, standardization efforts, and cultural dissemination. In terms of evidence generation, a large multicenter, placebo-controlled randomized clinical trial evaluated a standardized multi-herb formulation (Tongxinluo) and reported improved 90-day functional outcomes in acute ischemic stroke (61). In addition, rigorously designed trials are increasingly applied to TCM-related non-pharmacological modalities: a multicenter, sham-controlled randomized clinical trial reported that manual acupuncture as an adjunct to guideline-based care improved neurological function in acute cerebral infarction (72). Progress has also been made in establishing international standards for TCM, as evidenced by the publication of the TCM Clinical Practice Guidelines for Ischemic Stroke (83).

Further investigations have revealed that the internationalization trajectory of TCM is closely associated with cultural transmission. The philosophical foundations and therapeutic concepts of TCM have gained increasing acceptance worldwide, with modalities such as acupuncture and Tai Chi being widely adopted internationally (82). Additionally, the enhancement of global education and training systems in Chinese medicine has cultivated professionals equipped to support the international expansion of TCM (84). These developments collectively underscore the ongoing globalization of TCM in the context of ischemic stroke treatment.
